# Supplementary material for: Visual complexity of dental intake forms and its association with dental treatment outcomes: A retrospective cohort study
Source: PLoS One. 2025 Sep 4;20(9):e0331615. doi: 10.1371/journal.pone.0331615 (PMC12410745; doi:10.1371/journal.pone.0331615)
Supplement: S1 File — This script performs image processing to quantify the writing ratio of scanned or photographed patient intake forms. Implemented in Python (v3.9) with the OpenCV library (v4.5.5), the script converts each image to grayscale and applies binarization using Otsu’s thresholding method. The number of black pixels—representing printed elements and handwritten text—is calculated by subtracting the count of non-zero pixels from the total pixel count. The writing ratio is then computed as the proportion of black pixels relative to all pixels in the image. All images were processed under identical conditions to ensure consistency. (PDF) [file pone.0331615.s001.pdf]

```
import os
import csv
import cv2
import matplotlib.pyplot as plt

# Define the path to the dataset
dataset_path = "./datasets"

# Retrieve all .jpg files in the dataset directory
file_list = [f for f in os.listdir(dataset_path) if f.endswith(".jpg") and
not f.startswith(".")]

# Initialize a list to store output data
output = []

for i, filename in enumerate(file_list):
    # Read the image
    image = cv2.imread(os.path.join(dataset_path, filename))

    # Convert the image to grayscale
    gray = cv2.cvtColor(image, cv2.COLOR_RGB2GRAY)

    # Apply Otsu's thresholding to binarize the image
    _, bw_image = cv2.threshold(gray, 0, 255, cv2.THRESH_OTSU)

    # Calculate total number of pixels
    total_pixels = bw_image.size

    # Count the number of white pixels
    white_pixels = cv2.countNonZero(bw_image)

    # Calculate the number of black pixels
    black_pixels = total_pixels - white_pixels

    # Calculate the percentage of black pixels
    black_ratio = (black_pixels / total_pixels) * 100
```

```
    # Append the results to the output list
    output.append((i + 1, filename, total_pixels, white_pixels, black_pixels,
black_ratio))

# Write the results to a CSV file
with open(os.path.join(dataset_path, 'result.csv'), 'w', newline='') as f:
    writer = csv.writer(f)
    header = ["File No", "File Name", "Total Pixels", "White Pixels", "Black
Pixels", "Black Pixel Ratio (%)"]
    writer.writerow(header)
    writer.writerows(output)
```
