# Supplementary figures and images for: Visual complexity of dental intake forms and its association with dental treatment outcomes: A retrospective cohort study
Source: PLoS One. 2025 Sep 4;20(9):e0331615. doi: 10.1371/journal.pone.0331615 (PMC12410745; doi:10.1371/journal.pone.0331615)

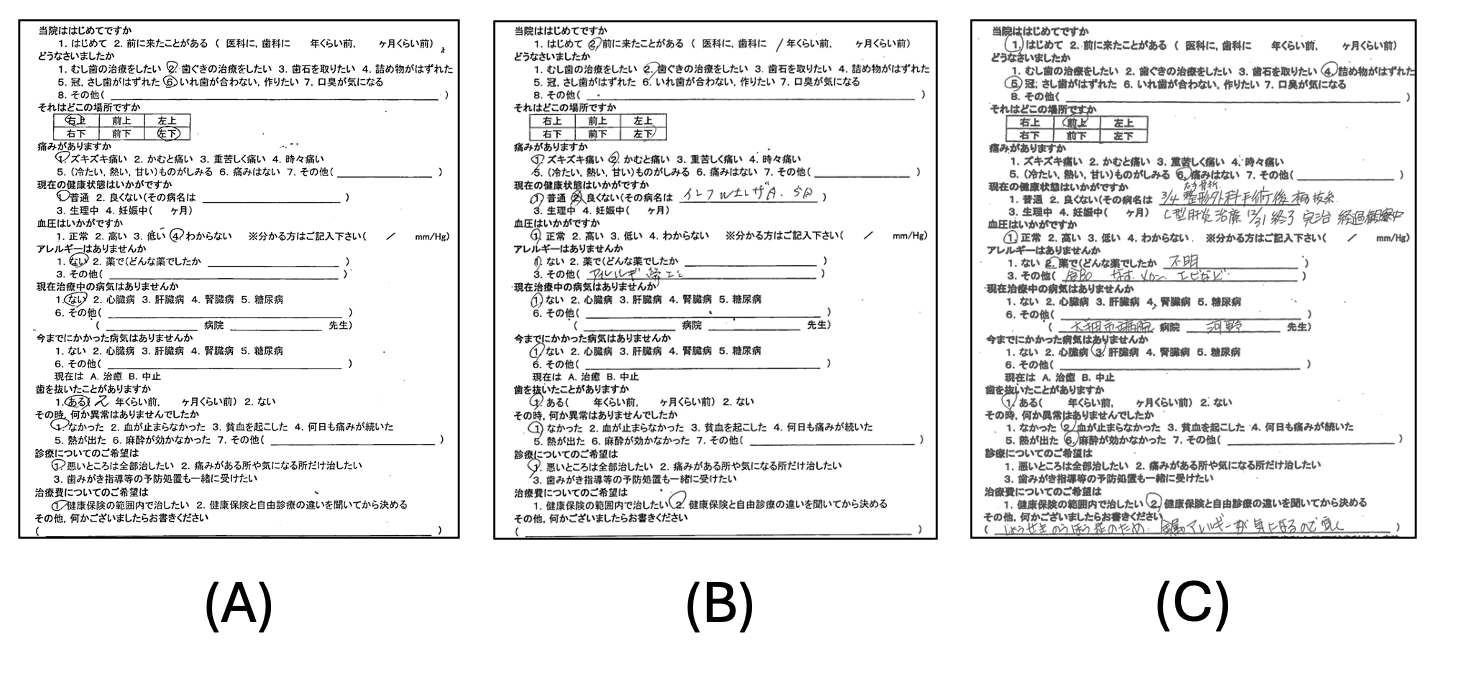

Supplement: S1 Fig — (A) Low writing ratio (5.4612%); (B) Mid writing ratio (8.4545%); (C) High writing ratio (10.5680%). These examples are scanned images of Japanese-language dental intake forms used in the present study. Each form includes structured items with checkboxes (e.g., current symptoms, medical history) and optional free-text sections. The amount of handwriting varies across groups, reflecting differences in patient engagement or communicative style. Personal information has been redacted. (TIFF) [file pone.0331615.s002.tiff]
