# Supplementary material for: Visual complexity of dental intake forms and its association with dental treatment outcomes: A retrospective cohort study
Source: PLoS One. 2025 Sep 4;20(9):e0331615. doi: 10.1371/journal.pone.0331615 (PMC12410745; doi:10.1371/journal.pone.0331615)
Supplement: Attachment — Submitted filename: Response to Reviewers.docx [file pone.0331615.s004.docx]

Reviewer #1: This would seem an interesting paper, covering some new aspects. Please see comments given below, and revise carefully.

Thank you for your encouraging remarks. We agree that the visual complexity of intake forms is an underexplored area in dental research, and we appreciate the opportunity to share our findings. We have carefully reviewed and addressed all specific comments provided, and we hope that the revised manuscript reflects the novelty and potential clinical relevance of our approach.

As a general comment, please study the Journal's Guidelines for Authors. Remember that your draft must be fully adapted to Journal style to be eligible to any proceeding.

Abstract

- With 244 words, this section does not provide maximum information. Remember that 300 words are allowed for with this section, so please add as much information as possible here. Please note that this section is most considered important to decide for switching to your full text.

Thank you for pointing out the word count limitation in the Abstract. We agree that the Abstract serves as a critical component in helping readers decide whether to engage with the full manuscript.

In response to your comment, we have revised and expanded the Abstract to include additional methodological details, a more nuanced summary of the results (including specific odds ratios and subgroup findings), and a more comprehensive conclusion. The revised Abstract now contains 284 words, offering a fuller overview of our study’s rationale, methods, key findings, and clinical implications while remaining within the 300-word limit.

We believe the updated version improves clarity and informational value, as you suggested. Thank you again for your constructive feedback.

- With your Conclusions, please stick exclusively to your revised aims. Do not simply repeat your results here. Do not speculate. Instead, provide a reasonable and generalizable extension of your outcome.

Thank you for your valuable suggestion regarding the Conclusion section. In accordance with your advice, we have revised the Conclusion to better align with the stated aims of the study. Rather than repeating specific results or engaging in speculative interpretation, we now focus on providing a generalizable and objective summary of the study’s contribution.

Intro

- Please revise for sound spacebar use, see "(...) provider-side(1-3) and patient67 side(4-6) (...).". Again, stick to uniform Journal style, and revise thoroughly.

Thank you for pointing this out. We carefully reviewed the entire manuscript to ensure that all in-text references, citation brackets, and adjacent spacing are consistent with the PLOS ONE style guidelines. Specifically, we corrected missing or inappropriate spaces around citation numbers and terms (e.g., "provider-side (1–3) and patient-side (4–6)") to maintain uniform formatting throughout the manuscript.

- What about a sound rationale? Please revise carefully.

We appreciate this important comment. We have revised the final part of the Introduction to more clearly state the rationale for our study. Specifically, we now highlight that examining the visual structure of intake forms addresses an overlooked aspect of patient behavior that may be visible even before clinical interaction begins. We also explain how this perspective not only aims to fill an existing gap in dental risk assessment but may also provide practical insights to improve the design of future intake forms. We believe this addition strengthens the justification for our research focus and clarifies its potential clinical relevance.

- "Specifically, we asked whether the appearance of a patient's intake form -quantified (...)." What about a sound and valid null hypothesis?

Thank you for highlighting this important point. We have revised the Introduction to explicitly state the null hypothesis: that there is no association between the visual characteristics of the intake form and dental treatment prognosis. We believe this clarifies the testable premise of our study.

Meths

- Details or references must be provided with your "applicable institutional guidelines" and "regulatory requirements".

Thank you for this helpful comment. We have revised the Materials and Methods to explicitly state that the study was conducted in accordance with the Declaration of Helsinki and approved by the Fukuoka Dental College Ethics Committee (approval no. 541). This clarifies the applicable institutional and international ethical guidelines.

- "Python (v3.9) and the OpenCV (v4.5.5)" - more details must be given here. With ALL materials and methodologies (including statistical software), please use general names with your text, followed by (brand name; manufacturer, city, ST[ate - abbreviated, if US], country) in parentheses. Stick to semicolon. Remember that reproducibility must be ensured.

Thank you very much for your valuable feedback regarding the description of software and libraries for reproducibility. We have revised the manuscript to include full details according to your recommendation. Specifically, we have updated the Image Processing and Quantification of Questionnaire Filling Area in the Methods section as follows:

“To quantify the amount of writing on the patient questionnaires, all image processing tasks were performed using Python (Python Software Foundation; Wilmington, DE, USA) version 3.9 and the OpenCV library (OpenCV.org; Palo Alto, CA, USA) version 4.5.5. Each scanned or photographed questionnaire image was first converted to grayscale, followed by binarization using Otsu’s thresholding method (cv2.threshold). The number of black pixels was then calculated using cv2.countNonZero and subtracted from the total pixel count to estimate the amount of written content, represented as a proportion of all pixels. All images were processed under identical conditions. A detailed algorithm is provided in Supplementary1.”

- Do not use legal terms with your full text. Delete "Inc.", "Corp.", and so on.

Thank you for your comment. We have revised the Materials and Methods section to remove the term “Corp.” and have updated the company name to “IBM” in accordance with the journal style guidelines.

Results

- Please add statistical tests with your p values.

Thank you for your valuable feedback. We have now added the specific statistical tests used for each p-value throughout the Results section.

- Provide representative "photographed questionnaire images".

Thank you for your valuable comment. As requested, we have added representative scanned images of intake forms to the Supporting Information section. These images illustrate examples from each writing ratio group (Low, Mid, High), with all personal identifiers fully redacted to ensure confidentiality.

Please refer to Supplementary 2 for these representative images. We have also cited this figure in the Results section for clarity.

- The readers surely would like know what you mean when referring to "specifically the writing ratio".

Thank you for pointing this out. We have revised the manuscript to clarify the definition and calculation of the “writing ratio,” including its rationale and technical details. The revised text now reads:

“The writing ratio was defined as the percentage of black pixels (representing intake form itself and handwriting) relative to the total pixel count of the form. This writing ratio serves as a quantitative proxy for the visual complexity of the intake form — capturing how much content, whether typed or handwritten, appears on the form regardless of its semantic content.”

Disc

- Stick to H0 when starting this section.

Thank you for your suggestion. In response, we have revised the beginning of the Discussion section to clearly state the null hypothesis tested in this study. The revised text now reads:

“This study was designed to test the null hypothesis that the visual complexity of dental intake forms—quantified by the writing ratio—has no association with treatment outcomes. We focused on whether this structural characteristic, independent of the content itself, could serve as an early behavioral signal related to patient prognosis.”

This change clarifies the study’s hypothesis and provides a stronger conceptual foundation for interpreting our results. We have also revised the following paragraphs to improve logical flow and avoid repetition.

- What about the strengths of your study?

Thank you for pointing this out. In response, we have added a paragraph in the Discussion section to highlight the strengths of our study. The revised section includes the following:

“This study has several strengths. First, it introduces a novel and objective metric—the writing ratio—to quantify the visual complexity of dental intake forms, offering a unique lens through which to understand early patient engagement. Second, the use of image-based analysis allowed for standardized and reproducible quantification across a large sample. Third, by applying multivariable regression and subgroup analyses, we were able to control for key confounding factors such as age, sex, and diagnosis, thereby strengthening the robustness of our findings.”

We believe that explicitly stating these methodological and conceptual strengths provides a clearer context for evaluating the contribution of our work.

- What about the limitations?

Thank you for your comment. We have now added a paragraph explicitly addressing the limitations of our study in the Discussion section, following the paragraph on study strengths and before the concluding remarks. This new section outlines several important limitations, including the retrospective design, the reliance on a single-center dataset, the lack of detailed sociodemographic data, and the limited generalizability due to potential institutional or cultural factors. We believe this addition provides a more balanced interpretation of our findings and clarifies the scope of applicability for our results.

- What about answers to your teaching objective?

Thank you for this thoughtful suggestion. We have added a new paragraph at the end of the Discussion section before the strength and the limitation of this study to explicitly address the potential educational implications of our findings. Specifically, we propose that recognizing visual cues in intake forms—such as sparse or disorganized writing—may enhance clinical education by encouraging students and clinicians to consider early behavioral signs of disengagement or psychological burden. This addition highlights how the study’s findings can inform teaching strategies in dental education and promote more patient-centered care from the initial encounter.

Concl

- Again, with your Conclusions, please stick exclusively to your revised aims. Do not simply repeat your results here. Do not speculate. Instead, provide a reasonable and generalizable extension of your outcome.

Thank you for your valuable feedback regarding the Conclusions section. In response, we have revised the conclusion to align more closely with the revised aims of the study. Specifically, we focused on whether the structural characteristic of intake forms—quantified by the writing ratio—is associated with dental treatment outcomes. The revised conclusion avoids speculative statements and does not simply repeat the results. Instead, it presents a generalizable interpretation of our findings, emphasizing their potential relevance for clinical risk identification and intake form design. We hope this addresses your concern.

Refs

- Please adapt thoroughly to uniform Journal style.

Thank you for your comment. We have carefully revised all references to ensure they fully comply with the PLOS ONE journal style, including formatting of author names, journal titles, volume and issue numbers, page ranges, and DOI information where applicable.

Reviewer #2: Overall Comments:

The authors have done a good job overall, but the manuscript needs major revisions to improve clarity and flow. Some parts are confusing or feel disconnected. It would help to rewrite the paper with a clearer structure and storyline. The analysis section especially needs more explanation — it should clearly state what was analyzed and why.

We sincerely appreciate the reviewer’s constructive summary and thoughtful suggestions regarding the clarity, structure, and flow of the manuscript. In response, we have thoroughly revised the manuscript to improve coherence and readability throughout. We have reorganized several paragraphs, clarified the rationale and hypotheses in the Introduction, and improved the explanation of our statistical analysis in the Methods and Results sections. In particular, we now more clearly specify the variables included in the logistic regression model, explain the rationale for our categorizations (e.g., age and writing ratio). We believe these changes have significantly improved the manuscript’s structure and overall clarity, and we thank the reviewer for prompting these important revisions.

Specific Comments:

1. Lines 71–72: The sentence is unclear and should be rewritten for clarity.

Thank you for pointing this out. We have revised the sentence to improve clarity and ensure accurate expression of the interaction between provider-side and patient-side factors. The revised sentence reads:

"The interaction between provider-side and patient-side factors—particularly in terms of communication, trust, and the quality of the patient-dentist relationship—is also crucial for successful treatment outcomes."

2. Lines 82–93: These statements need to be supported by appropriate references.

Thank you for your comment. The paragraph in question was intended to describe clinically observed patterns rather than to cite specific empirical studies. We have revised the wording to clarify that these statements reflect theoretical considerations and clinical impressions, not findings from prior literature. We have also softened the language and added a qualifying clause to make this distinction explicit in the manuscript.

3. Line 110: Please clarify this line.

Thank you for pointing this out. We have revised the sentence to clarify that the preliminary analysis relied on OCR technology to extract text from scanned intake forms and count the number of characters as a proxy for the amount of writing. This method was later replaced with an image-based approach due to limitations in OCR accuracy.

4. Why was data extracted in 2022 for the 2016 fiscal year? Please explain the rationale.

Thank you for your question. Data from the 2016 fiscal year were extracted in 2022 to ensure that each case had a minimum follow-up period of over five years. This time interval was necessary to allow for sufficient observation of treatment progress, completion, or dropout over the long term.

5. Line 186: The statement that there are 288 patients in each group is incorrect. Please revise and ensure accuracy.

Thank you for pointing this out. We have corrected the inaccurate statement regarding group sizes. The number of patients in each writing ratio group has been updated to reflect the actual tertile-based distribution: Low (n = 271), Mid (n = 270), and High (n = 272), as now stated in the manuscript.

6. The mean age of participants is nearly identical across groups. Was this by chance, or was it an intentional aspect of the study design? Please clarify.

Thank you for your comment. The similarity in mean age across the Low, Mid, and High writing ratio groups was not intentional, but rather the result of natural distribution during the tertile-based classification. We did not stratify or match participants by age when grouping by writing ratio.

7. Writing Ratio (8.76%), please confirm whether this value relates to the high or medium writing ratio category. As written, it belongs to both.

Thank you for pointing out the ambiguity regarding the writing ratio category. We have revised the manuscript to clarify the cutoff values for each tertile. The Mid group now clearly refers to patients with a writing ratio ≥7.8900% and <8.7626%, and we have adjusted the text accordingly:

“Patients were grouped into tertiles based on the writing ratio: Low (< 7.8900%, n = 271), Mid (≥7.8900 and <8.7626%, n = 270), and High (≥ 8.7626%, n = 272).”

This revision ensures precise classification and eliminates any overlap between categories. The figure and table labels have also been updated to reflect this correction.

8. Table 1: Consider adding variable names such as treatment outcomes and diagnoses, then the subcategories

Thank you for the helpful suggestion. We have revised Table 1 to include variable names such as Treatment Outcome and Diagnosis, followed by their respective subcategories. This change improves clarity and helps readers understand the structure of the table more easily.

9. Is diagnosis mutually exclusive? Can a single patient have more than one diagnosis? If so, please state this clearly in the manuscript.

Thank you for pointing this out. As diagnoses were not mutually exclusive, a single patient could have multiple diagnosis categories assigned. We have clarified this point in the manuscript by adding the following sentence to the Methods section:

“Diagnoses were not mutually exclusive; patients could be assigned to more than one diagnostic category based on their clinical presentation.”

10. In Table 1, please clarify what “Ratio (Mean ± SD)” refers to.

Thank you for pointing this out. We have clarified that “Ratio (Mean ± SD)” refers to the writing ratio, defined as the proportion of black pixels in the scanned intake form image. To enhance clarity, we have updated the label to “Writing Ratio (Mean ± SD)” in Table 1 and added a corresponding explanatory footnote.

11. I strongly recommend including age as a categorical variable in the analysis, rather than reporting only the mean.

Thank you for this important suggestion. We have revised the analysis by treating age as a categorical variable, rather than a continuous one. Specifically, age was divided into three categories: <50 years (reference), 50–64 years, and ≥65 years. The multivariable logistic regression model was updated accordingly, and the revised results are now shown in Table 2. The table clearly presents the odds ratios and p-values for each age group, with the reference category indicated. This change has also been reflected in the “Statistical Analysis” section of the manuscript. We believe this approach improves interpretability and aligns more closely with clinical reasoning.

12. Line 188: This appears to be a figure title. Please begin the following sentence on a new line. Also, results from the figure should be described in the text. (Note: the figure is unclear and difficult to interpret in its current form.)

Thank you for pointing this out. We have revised the figure title to ensure correct formatting and started the following sentence on a new line. As for the figure results, we had already described the distribution in the main text: “The writing ratio ranged from 5.46% to 11.63%, with a slightly right-skewed distribution (Fig 1).” We believe this satisfies the request for including figure results in the narrative. We have clarified the sentence formatting as requested.

Moreover, we have updated the figure itself to enhance readability by adjusting the layout, increasing label clarity, and improving contrast. The revised figure is now more easily interpretable and better aligned with the accompanying text.

13. “Outcome Distribution in Excluded Cases.” This section is unnecessary and should be removed.

Thank you for your suggestion. In accordance with your recommendation, we have removed the section titled “Outcome Distribution in Excluded Cases” from the manuscript.

14. Figure 2: The Y-axis should range from 0 to 100%. Also, clearly define what is meant by low, medium, and high writing ratios.

Thank you for your comment. We have revised Figure 2 so that the Y-axis ranges from 0 to 100% to improve clarity and consistency. We have also updated the figure legend to clearly define the thresholds used for the Low (<7.8900%), Mid (≥7.8900 and <8.7626%), and High (≥8.7626%) writing ratio groups.

Additionally, we clarified in the legend that the writing ratio is a proxy for the amount of content written by the patient on the intake form, representing initial engagement or communicative style.

15. Please consistently refer to and define what constitutes a poor outcome to maintain reader clarity.

Thank you for your helpful suggestion. We have revised the manuscript to ensure that the definition of a poor outcome—treatment dropout or clinician-initiated discontinuation—is stated clearly and consistently throughout the text.

16. It is sufficient to round to the values to the nearest hundredth, other than P values.

Thank you for your comment. As suggested, we have rounded most numeric values (e.g., means, standard deviations, odds ratios, and confidence intervals) to the nearest hundredth throughout the manuscript.

However, for the writing ratio thresholds used to classify patients into tertiles, we retained four decimal places (e.g., 7.8900%, 8.7626%) to ensure clarity and reproducibility of the categorical grouping. We believe this level of precision is necessary for accurate interpretation of the analysis.

17. In table 2, please explain why the "mid" ratio group was chosen as the reference group in the regression model.

Thank you for your insightful comment. We selected the Mid writing ratio group as the reference category in the regression model because it represents the intermediate range of the cohort, allowing for a balanced comparison between the extremes.

Using the middle tertile as the reference group enabled us to examine whether patients with particularly low or high visual complexity in their intake forms differed in treatment outcomes from the typical or average range. This approach also avoids setting either extreme as the normative baseline, which could introduce interpretive bias. We added this explanation to the results section.

18. All variables included in the regression model should be reported, whether or not they were statistically significant.

Thank you for your important suggestion. In response, we have revised Table 2 to include all variables used in the multivariable logistic regression model, including diagnosis categories, regardless of statistical significance. We have also added a clarifying footnote to Table 2 that states:

“All variables included in the logistic regression model—writing ratio (categorical), age group, sex, diagnosis category, and the experience level of the most senior attending dentist—are reported, regardless of statistical significance.”

19. Table 3: Clarify what "<50" refers to. Please mention the exact age of the youngest participant.

Thank you for your helpful comment. We have clarified that the "<50" age group refers to participants aged 13 to 49 years, with the youngest participant in the dataset being 13 years old. This clarification has been added to the footnote of Table 3. Additionally, we have included the age range (13 to 99 years) in the Results section to enhance clarity for readers.

20. Consider including an interaction term between age and sex in the regression model. Again, reporting age categorically rather than as a mean would be more informative.

Thank you for your suggestion. We agree that age and sex may interact in influencing treatment outcomes. However, given the exploratory nature of our study and the limited number of events in certain subgroups, we decided not to include an interaction term between age and sex in the current logistic regression model to avoid overfitting. Instead, we reported age as a categorical variable and conducted subgroup analyses that stratified patients by age and sex, which helped highlight potential patterns without inflating model complexity.

21. “Experience of Most Senior Dentist” This variable appears only in the regression model and is not introduced or discussed elsewhere. Values of this variable need to be reported before the regression model.

Thank you for your valuable comment. We have now added descriptive statistics for the variable “experience of the most senior attending dentist” to the Results section. Specifically, we reported the mean (12.78 years), standard deviation (8.72), and range (0 to 37 years) across the cohort.

We have added descriptive statistics for the variable “Years of experience of the most senior attending dentist” to Table 1. This provides readers with a clearer overview of all variables used in the regression analysis. A corresponding footnote has also been added for clarity.

22. Title "Supplementary Findings": Consider rephrasing this title to better reflect the content.

Thank you for your helpful suggestion. We have revised the section title from “Supplementary Findings” to “Supplementary Analysis: Characteristics of Patients with Extremely High Writing Ratios” to more accurately reflect the content and analytical focus of this section.

23. "Although the overall writing ratio was not significantly associated with poor outcomes in the High Ratio group" — please clarify the meaning of this statement.

Thank you for pointing this out. We have revised the sentence for clarity. Our intended meaning was that, while the overall multivariable analysis showed a significantly higher risk of poor outcomes in the Low writing ratio group, the High writing ratio group did not show a statistically significant association with poor outcomes when compared to the Mid group. We have clarified this wording in the supplementary section accordingly.

Additionally, in accordance with your former suggestion, we clarify the definition of poor outcome.

24. Please explain how you calculated the numbers 114 and 1028, given that the total sample size is 813. The sentence in question is:

25. "Among the top 10% of patients based on writing ratio (n = 114), 2.6% (3/114) had a phobia-related diagnosis. In contrast, only 0.8% (8/1028) of the remaining patients were similarly diagnosed."

Thank you for this important clarification request. In the revised analysis, we recalculated the “top 10%” group based solely on the final analysis cohort of 813 patients. As a result, the top 10% now includes 81 patients, of whom 3 (3.7%) had a phobia-related diagnosis. The remaining 732 patients included 7 (1.0%) such cases. This correction has been reflected in both the manuscript text and the updated figure. The previously stated numbers (114 and 1028) were based on the full intake population prior to applying exclusion criteria, which may have introduced confusion. We apologize for the oversight and have now ensured consistency throughout.

26. I expected to find an example of the intake form to better understand what information patients are asked to write. Please consider including one.

Thank you for your suggestion. To enhance clarity and allow readers to better understand the type of information patients are asked to provide, we have added representative examples of intake forms from each writing ratio group (Low, Mid, and High) as Supplementary Figure 1. These examples illustrate the visual differences in how patients complete the form and help contextualize our analysis of writing ratio.

Although the original intake forms are written entirely in Japanese, we have included brief explanations in the figure caption to indicate the general content of the forms—e.g., current symptoms, medical history, and other relevant patient information—to aid international readers in understanding the context.

27. Lines 297–301: There is repetition in these lines. Please revise for conciseness.

Thank you for pointing out the redundancy. We have revised the description of Fig 4 in the Results section to eliminate repetition and improve clarity. The revised text now reads:

“Fig 4. ROC curve of the exploratory scoring system. Receiver operating characteristic (ROC) curve evaluating the scoring system’s ability to identify patients at risk of poor outcomes. Using a cutoff of ≥3 points, the area under the curve (AUC) was 0.542 (p = 0.040), with sensitivity of 33.3%, specificity of 75.2%, and a Youden Index of 0.084, indicating modest discriminative performance.”

28. I would suggest replacing "not more likely" with "less likely."

Thank you for the suggestion. We agree that “less likely” offers greater clarity than “not more likely” in this context. The sentence has been revised accordingly to enhance readability and precision.

29. It's essential to highlight that this study analyzes the amount of writing in the intake form, regardless of content quality or completeness. Some patients may write less but provide concise and complete information, while others may write more but omit key details. This should be explicitly stated in the manuscript.

30. Please include the limitations of your study in your discussion.

Thank you for your thoughtful comment. We fully agree that the quantity of writing does not necessarily reflect the quality or completeness of the content. In response, we have explicitly acknowledged this limitation in the revised manuscript. Specifically, we now state that while the writing ratio offers an objective measure of visual complexity, it does not capture the semantic clarity or clinical adequacy of the written content. This has been addressed in the Limitations section of the Discussion.
